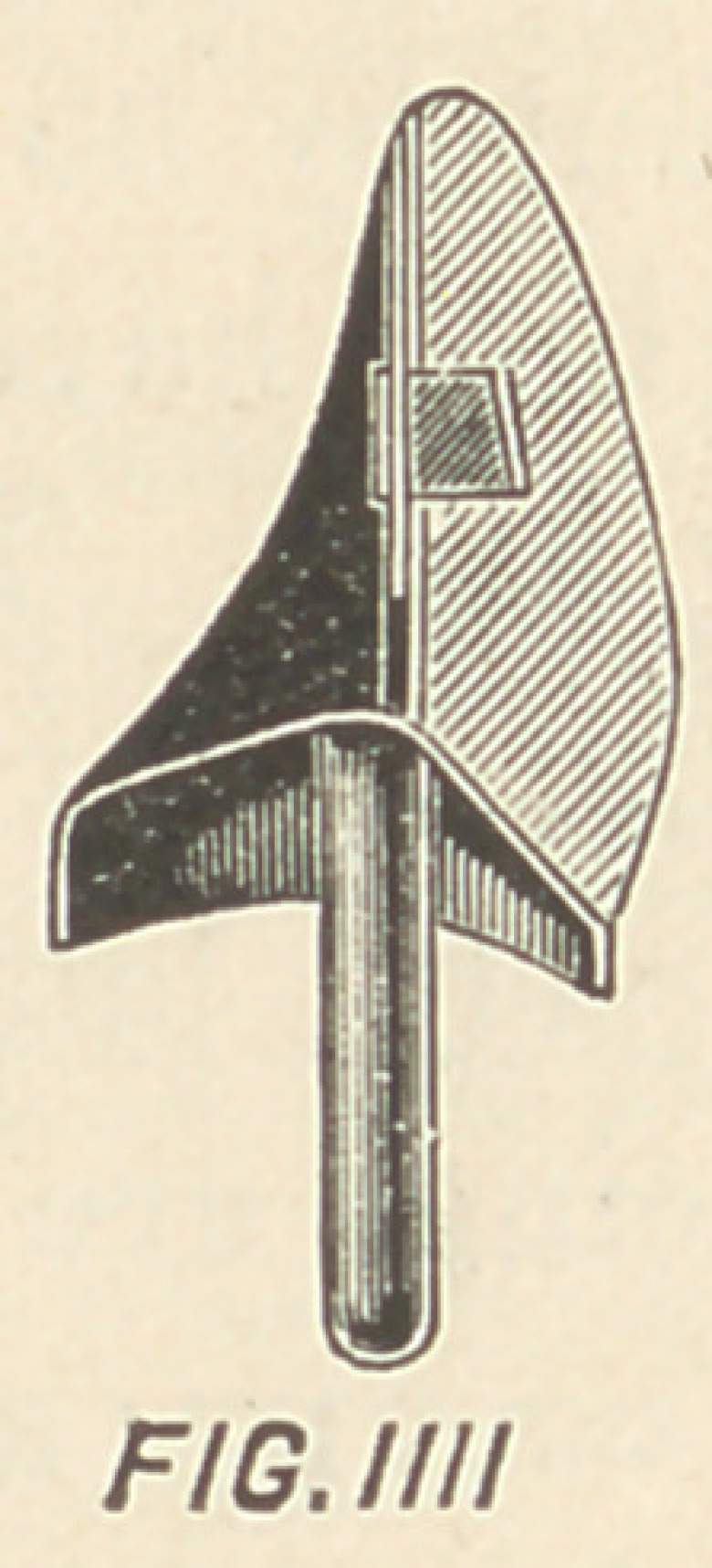# New Removable Facing

**Published:** 1900-06

**Authors:** Cephas Whitney

**Affiliations:** Jamaica, W. I.


### International Dental Journal.

Vol. XXI. June, 1900. No. 6.

### Original Communications.[1]

### NEW REMOVABLE FACING.

BY CEPHAS WHITNEY, D.D.S., JAMAICA, W. I.

Mr. President and Members of the Academy of Stomatology,—The very kind and favorable manner in which the keyed tooth facing and backing (which I shall present for your consideration to-night) has been received by many of your prominent members, has emboldened me to accept Dr. McQuillen's invitation to bring it to your notice.

Preliminary to going into the merits of my device, it may be well to touch on the main reasons which have made a non-soldered, easily repaired facing desirable.

Porcelain is a necessary weakness in dentistry; though possessing nearly every other virtue, such as natural color, cleanliness, and hardness, its ultimate dental strength is very disappointing.

From an engineering point of view, porcelain teeth should be at least double the strength of the force that can be naturally brought to bear upon them. This force we know to be in the neighborhood of three hundred pounds in exceptional cases; therefore, a tooth,

[1] The editor and publishers are not responsible for the views of authors of papers published in this department, nor for any claim to novelty, or otherwise, that may be made by them. No papers will be received for this department that have appeared in any other journal published in the country.

to comfortably support or resist this strain successfully, should have a natural breaking-point of six hundred pounds.

Fortunately, three hundred pounds is far in excess of the power that is possible for the average person to exert, and what muscular force they are able, or, better still, do ordinarily bring to bear, is usually distributed over several teeth. These facts, taken in conjunction with the movement, or given in plate work, the protection afforded by thick metal backings and incisal and occlusal metal extensions, explains why porcelain facings stand as well as they do.

The usual method of testing porcelain teeth by the makers gives them an erroneously high breaking point of from forty to one hundred pounds. In their method, only the pins project through an opening in a metal plate and are grasped by the drawing-tongs of the dynamometer. You will perceive that by this plan every part of the lingual surface of the facing is subjected to an even strain, of practically no leverage on the pin portion of the tooth. This is wrong, as they should be tested incisionally in imitation of the natural strain.

I believe that I will be unchallenged when I state that soldering injures facings in strength, that they are, even at the best, far weaker than good engineering calls for, hence their constant fractures and the well-known need of an easily repaired satisfactory facing. I have found by testing that the average soldered facing will fracture at about eighteen pounds' pressure on the incisoral fifth; whereas my removable facings fracture at about twenty-five pounds, and the reason for this gain will be shown later on in this paper.

But the facility for quick and perfect repairs is only one of many points that recommend the tooth. I will now proceed to describe it by drawings and passing specimens among you; then I will, tersely as possible, sum up its good points.

Fig. I. is a perspective view of the lingual surface of the facing A, which is provided with a flat, broad eye.

Fig. II. is a perspective view of the labial surface of the backing B, in which 2 is a socket for the reception of the eye 1 on Fig. I. when A and B are brought together; 3 in Fig. II. is a broad, thin bore, which connects with 1 and 2, into which the key C, Fig. III., wedges, thus firmly locking together A and B.

Fig. IIII. is a vertical section taken substantially on the lines 4, 4, in Figs. I. and II.

The comforts and advantages to be derived from using this device are enumerated as follows:

Extreme simplicity and speed in assembling, as the operator does not have to stop to make backings or care for porcelain in investments while soldering or annealing. Actual proximal contact of facings is secured, no dark shadows or gold being visible. This is impossible with soldered facings. The color of facings remains

FIG I FIG. II FIG. III FIG. IIII

as selected, because they never undergo the ill effects of investing and soldering. Strength of facing remains unimpaired, as it has been found that no matter how skilfully soldered facings are handled, they test out weaker than similar ones that have not been through the baneful process. Checking, visible or invisible, from soldering, is naturally impossible with this facing. As polishing and mounting crowns and bridges can be done with facings detached, there is little risk of accidental fracture.

Where gutta-percha or other heat materials are used for mounting crowns and bridges, especially open-faced caps, great comfort is obtained, for the reason that metal work is heated and mounted minus the facings, which are in no case liable to injury through heat and strains in mounting.

Sanitary union by wax or other weak insoluble material between facings and backings is secured; mechanical precision in contact of surface between facings and backings. This one point is a great advance over the usual rough way, even if duplex backings are resorted to; ease of repair in case of fractured facings. Fifteen minutes should be all that is necessary, as it only means the removal of eye on backing, removal of key, grinding in duplicate facing and reinserting key. Consider your patient's nerves and time as well as your own in a case of this kind. Compare this point with the old way.

Providing any facing is not satisfactory in color or for other reasons, at any stage of the proceedings, even after all is mounted, it is an easy matter to substitute a suitable one. In grinding facing and backing to conform to the case in hand, the parts remain in close unison, because of mechanical lock, and do not slide annoyingly one upon the other. Interchangeability is guaranteed.

With this device it is possible when desired to fashion occlusal and incisal protection extensions, and simply forming a nearly rectangular hole through the same for key, with a small fissure bur.

The last, but by no means smallest, point lies in the strength of the facings, which have been found by dynamometric tests to stand comparatively high. This virtue lies in the peculiar thin triangular form of the insert web, which is only twelve one-thousandths thick transversely, but extends longitudinally sufficiently far for strength. Facings are subjected to transverse fractures, and cross-pins usually occupy at least sixty one-thousandths space; even longitudinal pins take up thirty one-thousandths transverse room, whereas the rib in this tooth only weakens the tooth transversely by twelve one-thousandths.

## Figures and Tables

**FIG I f1:**
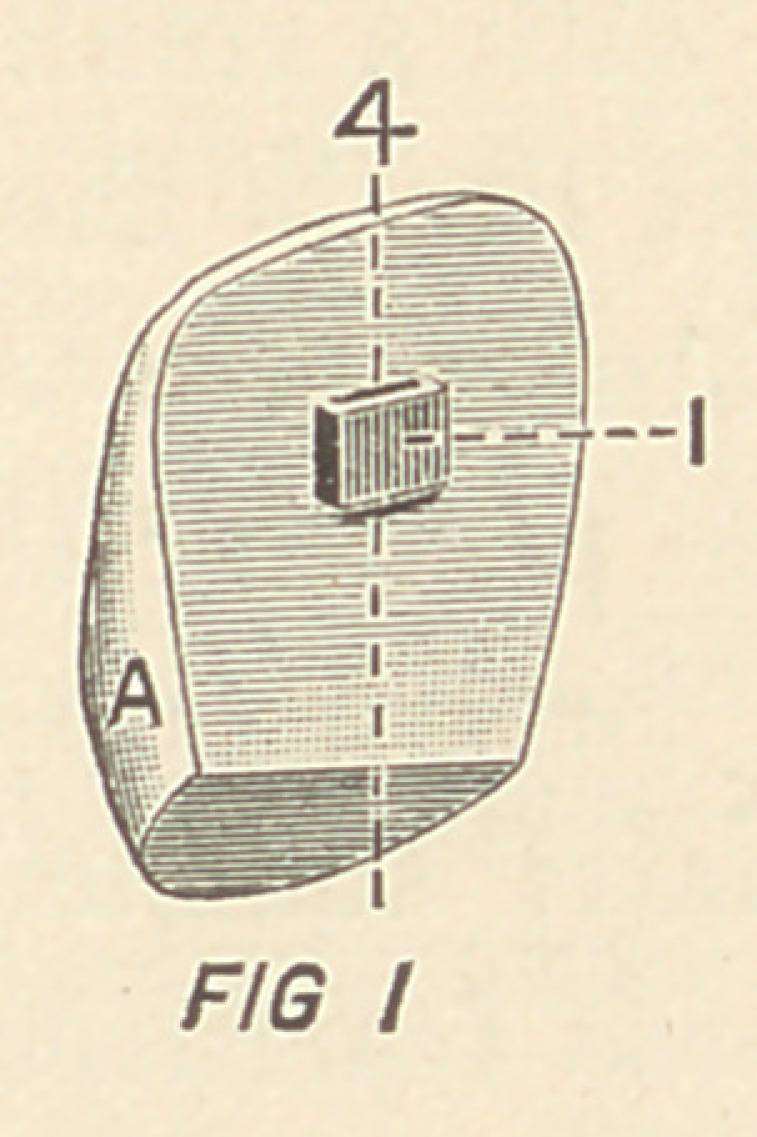


**FIG. II f2:**
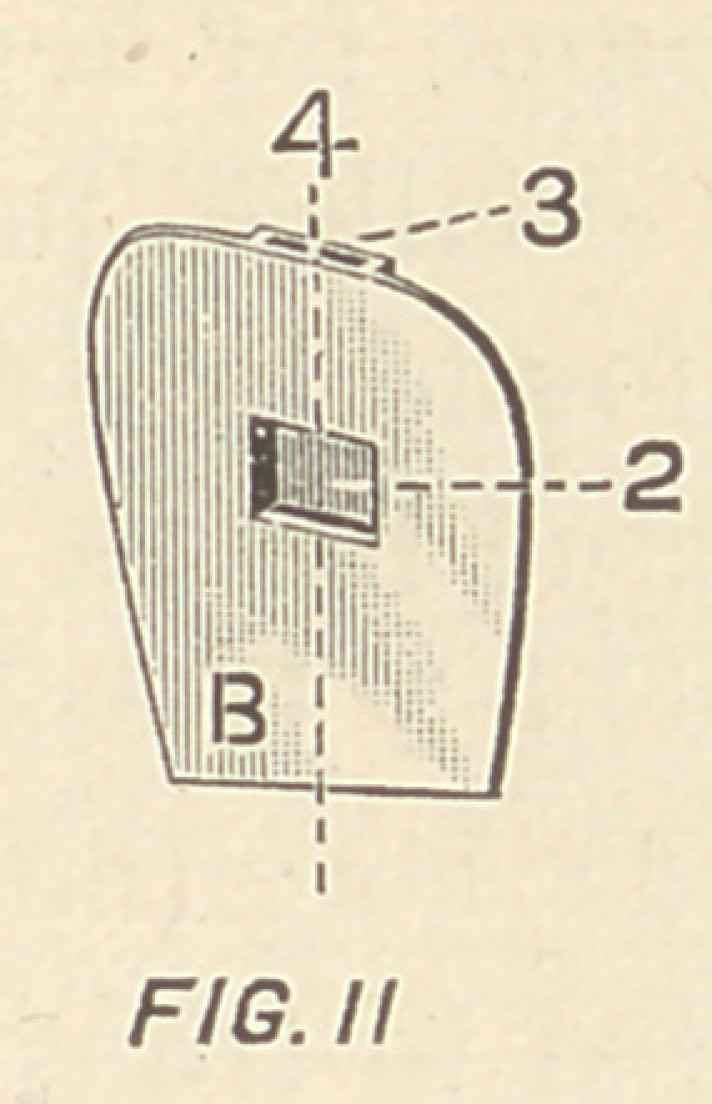


**FIG. III f3:**
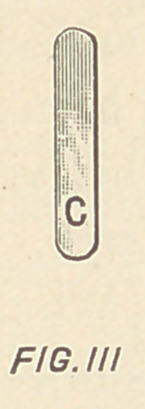


**FIG. IIII f4:**